# Meiotic Status Does Not Affect the Vitrification Effectiveness of Domestic Cat Oocytes

**DOI:** 10.3390/ani10081371

**Published:** 2020-08-07

**Authors:** Natalia Sowińska, Jennifer Zahmel, Wojciech Niżański, Romy Hribal, Lorena Fernandez-Gonzalez, Katarina Jewgenow

**Affiliations:** 1 Department of Reproduction, Poznan University of Life Science, ul. Wołyńska 35, 60-637 Poznan, Poland; 2 Department of Reproduction Biology, Leibniz Institute for Zoo and Wildlife Research, Alfred-Kowalke-Straße 17, 10315 Berlin, Germany; zahmel@izw-berlin.de (J.Z.); romyhribal@gmail.com (R.H.); fernandez@izw-berlin.de (L.F.-G.); jewgenow@izw-berlin.de (K.J.); 3 Department of Reproduction and Clinic of Farm Animals, Wroclaw University of Environmental and Life Science, Pl. Grunwaldzki 49, 50-366 Wroclaw, Poland; wojciech.nizanski@upwr.edu.pl

**Keywords:** cryopreservation, toxicity assessment, cryotop, Felidae, ICSI, embryo, morula, low efficiency

## Abstract

**Simple Summary:**

Assisted reproduction techniques (ART) are crucial for preserving endangered animal species. Cryopreservation by vitrification can maintain gamete viability for a long time. Efforts to preserve endangered species within the Felidae family are focused on developing appropriate ART procedures. The domestic cat is a good biomedical model. Unfortunately, the current state of knowledge on vitrification of cat oocytes is inconclusive and the efficiency of ART procedures is low. A key example concerns how the meiotic status of the oocyte influences suitability for vitrification. This is the main question of this study. First, we conducted a toxicity test to make sure that the vitrification solution (VS) we proposed does not have a toxic effect on cat oocytes. Next, we performed vitrification on cat oocytes before (nonmature) and after in vitro maturation (IVM) and checked their developmental potential. There was no negative impact of the applied VS on oocyte maturation and fertilization, demonstrating a possibility to obtain embryos in vitro regardless of the meiotic status. There is a need for more research on vitrification of the domestic cat oocytes as a model species for wild cats.

**Abstract:**

Cryopreservation is important for animal fertility and biodiversity. Unfortunately, cryopreservation of feline oocytes is still an experimental technique. The aims of this study were to analyze the potential toxicity of the cryoprotectants in the vitrification solution (VS) on cat oocytes and to investigate whether the meiotic status of oocytes influences their developmental potential after vitrification. Two experiments were conducted with the VS composed of 20% ethylene glycol, 20% dimethyl sulfoxide, 20% fetal calf serum, 1.5 M trehalose, and 10% Ficoll PM-70: (1) toxicity assessment of the VS on immature cumulus oocyte complexes (COCs), and subsequently in vitro maturation (IVM) and in vitro fertilization; (2) assessment of the influence of the meiotic status on vitrification effectiveness, where immature and in vitro matured COCs were vitrified on the Cryotop. After rewarming, vitrified oocytes were subjected to IVM (immature) and intracytoplasmic sperm injection (ICSI) with fresh epididymal sperm. The toxicity test revealed no negative effect of oocyte exposure to the applied VS on their developmental potential (*p* > 0.05). Although the vitrification procedure itself significantly reduced the meiotic competence of oocytes, their meiotic status before vitrification (immature vs. in vitro matured) did not influence fertilization and morula rates. The only parameter affected by vitrification was the rate of oocytes suitable for ICSI, which was significantly lower for immature oocytes. Regardless of the meiotic status of vitrified oocytes, morphologically normal morulae were obtained. Moreover, the two meiotic stages examined are suitable for vitrification, with mature oocytes being a better choice when a well-equipped laboratory is available.

## 1. Introduction

Cryopreservation of female gametes is a crucial part of assisted reproductive technologies (ART) because it eliminates the obstacles of time and distance between gamete collection and fertilization. With dramatic declines in populations of threatened cat species, oocyte cryopreservation is an extraordinarily crucial issue that could help increase the number of individuals and genetic diversity. Domestic cats are a model animal for nondomestic cat species, but despite dynamic development of cat ART in recent years, significant protocol improvement is still necessary. One of the primary issues that needs to be addressed concerns the meiotic stage of oocyte appropriate for cryopreservation. 

Three methods of gamete cryopreservation have been applied to cats: slow freezing, ultrarapid freezing, and vitrification. For feline sperm cells, cryopreservation is a well-established procedure. Banked samples of frozen semen are available, at least for in vitro fertilization (IVF) or intracytoplasmic sperm injection (ICSI) [1]. Nevertheless, only a few studies reported fertilization of cryopreserved cat oocytes and in all cases, results were less satisfactory than in ungulates or rodents [2,3,4,5,6]. Generally, cryopreservation of immature or in vitro matured cat oocytes is considered a non-reliable and non-repeatable method. 

Vitrification in reproductive biology was reported for the first time in 1985 by Rall and Fahy [7]. It soon became the method of choice for cryopreservation of oocytes and embryos in humans and many animal species due to extremely high effectiveness [8,9]. The first successful vitrification of cat oocytes was performed by Murakami et al. [10] in 2004. In vitro matured oocytes were vitrified and, after thawing, subjected to in vitro fertilization by epididymal spermatozoa. The best results observed were 20% of cleaved zygotes (19/64) and 3.7% blastocysts (2/64). In another study on vitrification of in vitro matured oocytes, a fertilization rate of 16% (49/303) was reported [4]. Further published research has focused on vitrification of immature oocytes [2,3]. Comizzoli et al. [3] reported vitrification of immature cat oocytes exposed to resveratrol in order to compact the germinal vesicle. The highest cleavage rate of vitrified and fertilized oocytes achieved was 48.7% (29/61), but no morula or blastocyst were obtained.

A major obstacle in the application of vitrification is the potential toxicity of high concentration of cryoprotectants in vitrification solutions (VS). Various strategies have been described to reduce this risk including: (1) the use of cryoprotectant mixtures of reduced toxicity; (2) addition of non-permeating cryoprotectants such as disaccharides (e.g., trehalose or sucrose) or high molecular weight molecules (e.g., Ficoll or polyvinylpolypyrrolidone (PVP)), which can reduce the amount of permeable cryoprotectants needed for vitrification; (3) shortening the time of oocyte/embryo exposure to cryoprotectants [11,12,13]. In our previous experiment on optimizing the composition of vitrification solution, we applied all of the three strategies described above [14]. The following combination of procedures were the most effective: oocyte vitrification in VS consisting of 20% dimethyl sulfoxide (DMSO), 20% ethylene glycol (EG), 1.5 M trehalose, 10% Ficoll PM-70, 20% fetal calf serum (FCS), and application of a 3-step vitrification and warming protocol [14]. Thus, in the present study the above procedures were utilized.

Studies conducted on other species such as pig or cattle suggest the outcome of cryopreservation is influenced by the stage of meiotic maturation of oocytes [15,16], but no appropriate data is available for the vitrified oocytes of domestic cats [17,18]. Recently the cryosurvival rate of in-vitro matured oocytes immediately after thawing was reported to be >60%, while parthenogenetic activation of warmed vitrified oocytes resulted in a cleavage rate of 22% [19], whereas the IVM rate of immature cat oocytes after vitrification was reported by another author [20] to be about 20% and half of them cleaved after fertilization To our best knowledge, there is no published evidence comparing vitrification of immature and in vitro matured oocytes of this species under identical lab conditions. Therefore, we aimed to analyze the potential toxicity of the cryoprotectants included in the VS on cat oocytes (experiment 1) and to investigate whether the stage of nuclear maturation of oocytes (immature vs. in vitro matured) influences the oocyte suitability for ICSI and embryonic development following fertilization by ICSI (experiment 2). 

## 2. Materials and Methods

All chemicals were purchased from Sigma-Aldrich, unless otherwise stated.

### 2.1. Oocyte Collection and In Vitro Maturation (IVM)

Oocyte collection and IVM were performed according to a protocol described by Waurich et al. [21]. Briefly, ovaries from adult queens were collected after routine ovariohysterectomy from local veterinary clinics and transported to the laboratory within 4 h after surgery. Ovaries were stored in Minimum Essential Medium Eagle HEPES Modification supplemented with 3 mg BSA/mL and 1x Antibiotic Antimycotic Solution (A5955) and shipped in 50 mL Greiner tubes at 4 °C in styrofoam boxes. Ovaries were processed immediately upon arrival to the lab. Cumulus oocyte complexes (COCs) were obtained after slicing of the ovaries with scalpel blade and rinsing with washing medium (WM). The WM consisted of Medium 199 containing Earle’s salts, supplemented with 3 mg/mL BSA (Bovine Serum Albumin), 0.1 mg/mL cysteine, 1.4 mg/mL HEPES, 0.25 mg/mL sodium pryruvate, 0.6 mg/mL sodium lactate, 0.15 mg/mL L-glutamine, and 0.055 mg/mL gentamicin. Oocytes with a dark, homogenous cytoplasm, surrounded by several layers of compacted cumulus cells were selected. For in vitro maturation, 5–10 COCs were placed into 400 µL of maturation medium (MM), which consisted of WM supplemented with 0.02 IU/mL of FSH and 0.02 IU/mL of LH, covered with 400 µL mineral oil in a 4-well dish of Nunc^®^ Thermo Fisher Scientific. IVM was performed at 38.5 °C under 5% CO_2_ in a humidified air atmosphere for 24 h. Directly after maturation COCs were vitrified or fertilized (control group).

### 2.2. Sperm Isolation

Sperm cells were isolated according to a protocol described by Waurich et al. [21]. Testis with epididymis from adult domestic tom cats were collected after routine orchiectomy from local veterinary clinics and transported to the laboratory. Testes were stored in Greiner tubes at 4 °C and shipped to the laboratory in styrofoam boxes within 4 h after surgery. Testes were maintained without medium and stored for a maximum of 24 h at 4 °C. Epididymis’ tail were cut out from the testis and minced in 1 mL TALP (Tyrode’s salts solution supplemented with 6 mg BSA/mL, 1.2 mg HEPES/mL, 1.1 mg sodium lactate/mL, 0.15 mg L-glutamine/mL, and 0.1 mg sodium pyruvate/mL). After motility assessment, sperm cell suspension was centrifuged at 500× g for 5 min and the pellet was resuspended in 100 µL of medium. The final concentration was calculated utilizing a Neubauer Chamber (Brand GmbH + Co. KG, Wertheim, Germany) in accordance with the manufacturer’s instructions.

### 2.3. In Vitro Fertilization (IVF)

A concentration of 1 × 10^5^ motile sperm/mL was used. Sperm cells were co-incubated with 5–10 in vitro matured COCs for 16–18 h in 400 µL of TALP medium supplemented with 2.2 IU heparin, covered with 400 µL mineral oil in a 4-well dish of Nunc^®^ Thermo Fisher Scientific. IVF was performed at 38.5 °C under 5% CO_2_ in a humidified air atmosphere for 24 h.

### 2.4. Intracytoplasmic Sperm Injection (ICSI) 

All COCs subjected to ICSI were mechanically stripped off the cumulus cells by gentle pipetting. Only oocytes with a clearly visible polar body, tense and intact oolemma were chosen for ICSI. Oocytes with damaged, rough, or folded oolemma, as well as oocytes with severe mosaic transparency or ooplasm fragmentation were considered inappropriate for ICSI and excluded from further culture.

ICSI was performed at 38.5 °C under an inverted microscope (Axiovert 100, Carl Zeiss, Jena, Germany) according to the protocol previously described by Ringleb et al. [1]. Sperm solution was diluted 1:10 in 10% PVP medium (Gynemed GmbH, Lehnsan, Germany). A single motile sperm cell was immobilized by drawing an injection pipette (5 µm inner diameter, Gynemed GmbH) across the mid-piece. Sperm was then pulled into the pipette and injected headfirst from the 3 o’clock position while polar bodies were positioned at 6 or 12 o’clock.

### 2.5. Embryo Culture In Vitro (IVC)

Directly after IVF or ICSI, oocytes were transferred to embryo culture medium. Embryo culture was performed according to a protocol described by Comizzoli et al. [22]. Briefly, embryos were incubated in 400 µL Ham’s F10 (Modified Ham’s F10 Basal Medium; Irvine Scientific, Medical Technology Vertriebs GmbH, Bruckberg, Germany), supplemented with 5% FCS (vol:vol), 1 mM sodium pyruvate, 1 mM L-glutamine, 0.1 mg/mL streptomycin, and 100 IU/mL penicillin under 400 µL mineral oil. The IVC was carried out up to 9 days after IVF or ICSI.

### 2.6. Assessment of Oocyte Nuclear Maturation and Embryo Development

First, 48 h post IVF/ICSI, all presumptive zygotes were examined under an inverted light microscope and those that did not cleaved were mechanically stripped off the cumulus cells by gentle pipetting (post IVF) and fixed in a 2% formaldehyde solution for 15 min. Subsequently, the DNA of oocytes was stained with Hoechst 33,342 DPBS solution (2 µg/mL) for 10 min at room temperature. After staining, oocytes were washed three times in DPBS supplemented with 5% BSA. Oocytes were then mounted on glass slides, sealed under coverslips with Vaseline^®^ (Unilever, London, UK), and stored for a maximum of 48 h in a humid, dark chamber at room temperature until analysis under an epifluorescence microscope (Leica DMLB, Wetzlar, Germany).

**Figure 1:**
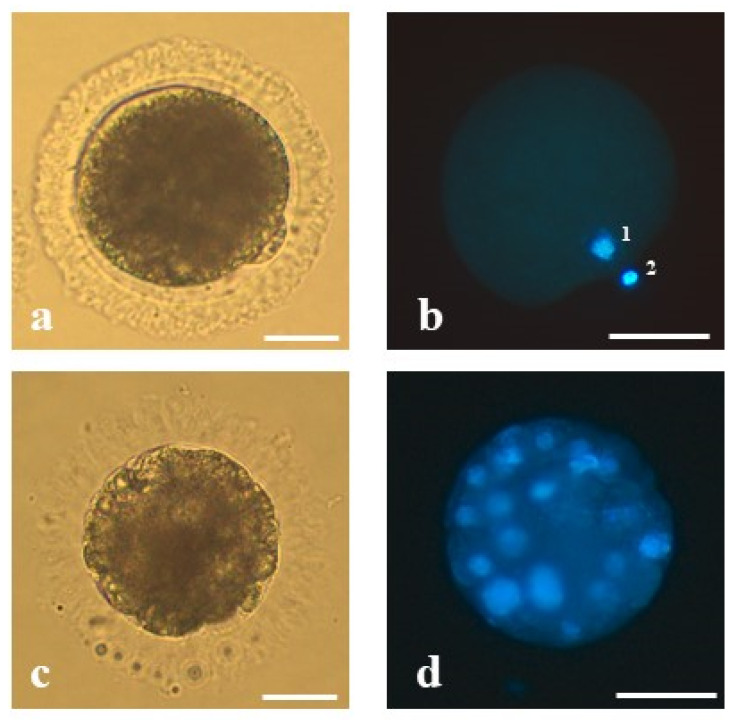
The representative oocyte and morula stage embryo of the domestic cat analyzed in the present study: (**a**) mature oocyte of proper morphology at the second metaphase stage with extruded first polar body, (**b**) mature oocyte stained with Hoechst 33,342—(1) visible chromatin of the oocyte and (2) the first polar body, (**c**) an embryo at the morula stage on 5 dpi derived from an oocyte matured in vitro, vitrified, fertilized by intracytoplasmic sperm injection and (**d**) stained with Hoechst 33,342. Bars represent 50 µm.

Meiotic stage of oocytes was classified based on the report of De los Reyes et al. [23] and Comizzoli et al. [3,24]. Chromatin configurations were classified as follows: germinal vesicle (GV)—nucleus with highly decondensed chromatin protected by an envelope; germinal vesicle break down; (GVBD)—the nuclear envelope breaks, the chromatin disperse, and condensation initiate; first metaphase (MI)—pairs of chromosomes (bivalents) arrange on the metaphase plate; and the second metaphase (MII)—chromosomes in the second metaphase plate, the first polar body (PB) extruded (Figure 1a,b). Meiotic competence was defined as the number of MII oocytes relative to the total number of oocytes in experimental group.

Embryo development was assessed every 48 h under an inverted light microscope up to 9 days post ICSI/IVF. Embryos that stopped in embryonic development were fixed, stained, and evaluated using the same procedure as for oocytes. Embryos were analyzed under an epifluorescence microscope (Leica DMLB, Wetzlar, Germany).

Cleavage rate was defined as percentage of cleaved zygotes (the presence of at least two blastomeres) and was calculated relative to the total number of oocytes subjected to ICSI/IVF. In the subsequent stages of embryonic development, the number of blastomeres was counted. Morula stage embryos were determined by the presence of at least 16 blastomeres (Figure 1c,d) and blastocyst stage embryos of at least 50 blastomeres [25]. Morulae and blastocyst rate were defined as the number of morulae or blastocysts produced relative to the total number of cleaved embryos.

### 2.7. Experimental Design

In Experiment 1, in order to test the toxic impact of cryoprotectants, we evaluated the exposure of immature oocytes to VS on in vitro maturation (IVM) and fertilization (IVF).

In Experiment 2, immature and in vitro matured oocytes were subjected to vitrification, and after rewarming, subjected to IVM (immature) and ICSI (both categories of oocytes) to verify their meiotic and developmental competences. The following five groups of feline COCs were included into this study: 

Experiment 1: cryoprotectant toxicity test. (1) Experimental—oocytes washed in the VS/warming solutions, matured in vitro, and subjected to IVF; (2) control group—oocytes matured in vitro and subjected to IVF.

Experiment 2: immature vs.in vitro matured oocytes. (1) Experimental 1—immature, vitrified, matured in vitro and subjected to ICSI; (2) experimental 2—in vitro matured, vitrified, and subjected to ICSI; (3) control group—oocytes matured in vitro and subjected to ICSI (non-vitrified).

#### 2.7.1. Experiment 1. Cryoprotectant Toxicity Test

For the toxicity assessment, 47 immature COCs were washed in VS and warming solutions (according to vitrification and warming protocol, but with omission of the liquid nitrogen (LN_2_ plugging). Then COCs were in vitro matured, in vitro fertilized (IVF), and in vitro cultured in order to assess the influence of vitrification/warming solutions on oocyte developmental competency. For control, oocytes (*n* = 46) were matured in vitro and subjected to IVF. Triplicate repeats for all oocyte groups were performed.

#### 2.7.2. Experiment 2. Cryotop Vitrification and Warming—Immature vs. In Vitro Matured Oocytes

Based on the published data, our preliminary studies, and the results of Experiment 1 we decided to use the vitrification solution (VS) consisting of 20% ethylene glycol (EG) 20% dimethyl sulfoxide (DMSO), 1.5 M trehalose, 10% Ficoll PM-70, and 20% FCS in Dulbecco’s Phosphate Buffered Saline (DPBS).

Immature or in vitro matured COCs were washed in WM and vitrified as follows: groups of two COCs were incubated for 3 min in 5% ethylene glycol (EG) and 5% DMSO in DPBS, followed by 3 min incubation in 10% EG and 10% DMSO in DPBS and immediately placed on a cryotop (Kitazato BioPharma Co., Ltd. Fuji, Shizuoka Japan) in a minimum volume (<0.5 µL) of VS (20% DMSO, 20% EG, 1.5 M trehalose, 10% Ficoll PM-70, and 20% FCS in DPBS). Within 30 s, COCs were plunged into LN_2_. All vitrification steps were performed at room temperature.

After at least 24 h of storage in LN_2_, vitrified COCs were warmed by plunging the cryotop in the warming solution 1 (WS1) for 30 s. WS1 consisted of 1 M trehalose and 30% Ficoll PM-70 in DPBS. Subsequently, COCs were incubated for 3 min in warming solution 2 (WS2), which consisted of DPBS supplemented with 0.5 M trehalose and 15% Ficoll PM-70. The whole warming process was performed at 38.5 °C. COCs were then transferred to WM, supplemented with 10% Ficoll PM-70, and kept for 2 h in an incubator (38.5 °C, 5% CO_2_) to allow recovery from cryopreservation before IVM (experimental group 1, *n* = 154) or ICSI (experimental group 2, *n* = 156) procedure. For control, oocytes were matured in vitro and subjected to ICSI (non-vitrified, *n* = 233).

### 2.8. Statistical Analysis

All experiments were replicated at least three times on different days with different batches of oocytes.

Data were compared with the chi-square test (Statistica for Windows, Stat Soft Inc., Tulsa, OK, USA). Probabilities of less than 0.05 (*p* < 0.05) were considered statistically significant.

### 2.9. Ethical Statement

Live animals raising ethical concerns were not used in this study.

## 3. Results

### 3.1. Experiment 1. Cryoprotectant Toxicity Test

None of the analyzed parameters (maturation, fertilization, and morula rates) were significantly (*p* > 0.05) affected by exposure to VS. Morula was the most advanced stage of development observed (Table 1).

### 3.2. Experiment 2. Cryotop Vitrification—Immature vs. In Vitro Matured Oocytes

Maturation rate significantly declined as a result of vitrification and ranged from 51.5% (120/233) for control oocytes to 14.9% (23/154) for vitrified oocytes. Only few of the IVM oocytes (10.4% 16/154) met the morphological requirements for ICSI, which contrasts the rate of control oocytes (49.4%, 115/233) subjected to ICSI (Table 2).

### 3.3. Developmental Competence of Vitrified Oocytes

ICSI was performed on 112 oocytes: 16 vitrified as immature and 96 vitrified after IVM. In the group of immature oocytes, four embryos (25%) were produced, including two 2-blastomere embryos, one 4-6-blastomere embryos, and one morula. With regard to IVM oocytes, 21 embryos were obtained (21.9%) including six 2-blastomere embryos, six 4-6-blastomere embryos, two 8-12-blastomere embryos, and seven morulae. No blastocyst was obtained from vitrified oocytes of any source, whereas 16 blastocysts were obtained in the control (non-vitrified) oocytes. Oocyte status before vitrification (immature vs. in vitro matured) did not influence fertilization and morula rates (*p* > 0.05), however the percentage of vitrified oocytes suitable for ICSI was significantly lower for immature oocytes. All measured parameters were significantly (*p* < 0.05) higher in control (non-vitrified) oocytes than in the two vitrification groups (Table 2). In addition, the cleavage rates were significantly reduced between the two vitrification groups if the cleavage rates were calculated based on the total number of oocytes used for the experiment (vitrified immature, vitrified mature, and non-vitrified—2.6%, 13.5%, and 28.3%, respectively).

## 4. Discussion

Improvement of protocols for long-term storage of oocytes is necessary to maximize the practical efficiency of in vitro protocols in the preservation of genetic materials of feline species [26]. This study investigated: (1) a possible toxic effect of vitrification solution (VS) consisting of 20% DMSO, 20% EG, 1.5 M trehalose, 10% Ficoll PM-70; 20% FCS and (2) influence of the oocyte meiotic status (immature vs. in vitro matured) on oocyte suitability for ICSI and on developmental competence of resulting embryos.

The toxicity test revealed no negative effect of oocyte exposure to the applied VS on their developmental potential. Although the vitrification procedure itself significantly reduced the meiotic competence of oocytes, their meiotic status before vitrification (immature vs. in vitro matured) did not influence fertilization and morula rates. The only parameter affected by vitrification was the rate of oocytes suitable for ICSI, which was significantly lower for immature oocytes.

### 4.1. Cryoprotectant Toxicity

Since the first application of vitrification to oocyte and embryo cryopreservation, the use of approximately 20 different combinations of cryoprotectants have been recorded. The number of possible variants of protocols considering cryoprotectants types, their concentrations, incubation times, and other conditions is almost infinite [13,27]. It has been shown in mice that high concentrations of permeable solution might be toxic to the oocyte [28]. In cats, information about the toxic effect of high concentrations of cryoprotectants combinations on oocyte quality is limited [29]. Comizzoli et al. [24] demonstrated that elevated concentration of EG and PrOH (3 M) disrupted the cytoskeleton of mature feline oocytes and their ability to develop up to blastocyst stage in vitro. Later on, Tharasanit et al. [29] showed that exposure to DMSO (20%), EG (20%), and sucrose (0.5 M) did not affect meiotic competence of GV oocytes compared with control gametes. Apart from these, the toxicity of different cryoprotectants and their effect on vitrification efficiency have not been tested in the cat. 

High concentrations of cryoprotectants are needed in a vitrification process to achieve solidification of the vitrification solution that contains an oocyte without ice nucleation and crystallization [27]. Thus, every vitrification protocol requires a high concentration (approximately 30–50%) of permeable cryoprotective agents, such as DMSO, EG, and PrOH [30]. The addition of non-permeating cryoprotectants such as disaccharides or high molecular weight molecules helps to reduce the potential harmfulness of the vitrification solution. The VS used in our study was comprised of 20% DMSO, 20% EG, 1.5 M trehalose, 10% Ficoll, PM-70, and 20% FCS [14]. The two components, disaccharide (trehalose) and high molecular weight polymer (Ficoll PM-70), are non-permeable cryoprotectants with no toxic effect on cells. They prevent formation of ice crystals by increasing the viscosity of vitrification solution. Our previous research shows that the addition of Ficoll PM-70 improves survival of cat oocytes upon vitrification [31]. The role of FCS was to minimize osmotic stress by controlling osmotic pressure indirectly through the albumin present in FCS [32].

In the present study, we assessed a possible toxicity of washing immature oocytes in the VS solution on their maturation and developmental capacity of resulting IVF embryos (Table 1). The toxicity test did not show any negative impact on in vitro maturation of oocytes and on developmental competence of resulting embryos. Thus, our vitrification solution can be safely used for further experiments.

### 4.2. Cryotop Vitrification and Warming—Effect of Meiotic Stage of Oocytes

It has already been shown that the ability of an oocyte to be fertilized and develop into an embryo depends on its meiotic competence and on cytoplasmic maturation [33]. Here the developmental competence of feline oocytes after vitrification and warming was analyzed in relation to its maturation stage before vitrification.

The theoretical discussion regarding the most appropriate stage of oocyte nuclear maturation for vitrification is as long as the history of gametes cryopreservation. The published evidence on which nuclear maturation stage is more sensitive to cryoinjuries are often contradictory or mutually exclusive. There are several aspects to consider when talking about the suitability of a particular maturation stage of an oocyte for vitrification including nucleus structure and chromatin arrangement, oolemma stability, influence of cumulus cells, osmotic response, selection conditions, equipment availability, as well as complexity and time requirements of the procedures [17,18,29,34,35]. 

It is known that immature oocytes at the germinal vesicle (GV) stage are characterized by a large diploid nucleus (prophase I), a dense band of filamentous actin subjacent the oolemma, and several other organelles scattered in the ooplasm, such as mitochondria, endoplasmic reticulum, and Golgi apparatus [34,36]. The nucleus of a mature oocyte (metaphase of the second meiotic division, MII) is characterized by a large, peripheral spindle apparatus with microtubules which formed barrel-shaped structures with slightly pointed poles by organized structures traversing from one pole to the other and haploid set of chromosomes arranged in a compact metaphase plate at the equator of the structure. Microfilaments are localized mainly in the cortex (disposed beneath the oolemma), overlying the metaphase chromatin and polar body [31]. In the MII oocyte, cortical granules migrate to the periphery of the ooplasm just beneath the actin band, where they are ready to undergo exocytosis at the time of fertilization [34].

Due to the deference in the nuclear organization, GV stage oocytes are considered to be more tolerant of cryopreservation. The chromatin of the GV oocytes is theoretically protected by the nuclear envelope and the cold-sensitive structure of the meiotic spindle has yet to be formed [29,35]. On the other hand, some authors postulate that chilling injuries are more frequent in GV oocyte, due to decreased cell membrane stability and due to the particular cytoskeletal form at this stage [37]. Moreover, immature oocytes are surrounded by several layers of compact cumulus cells that may be either a physical barrier against cryoprotectants [18] or an obstacle to water output [13]. It has also been reported that the vitrification process reduces cumulus cell viability [38] and affects gap junction communication between oocyte and cumulus cells [39]. This later on may affect an oocyte’s ability to mature. We confirmed this hypothesis by showing that only 14.9% of feline vitrified GV oocytes were able to mature in vitro (vs. 51.5% in the control group).

The role of cumulus cells during the process of oocyte vitrification is not entirely clear. It has been suggested that cumulus cells protect against rapid influx and efflux of cryoprotectants [40] and can physically protect oocytes during exposure to low temperatures [38]. Recent studies in sheep have provided evidence that removal of cumulus cells from immature oocytes before vitrification enhances oocyte survival and meiotic competence [41]. Several studies, including ours, showed that addition of fresh granulosa cells to IVM medium may improve meiotic and developmental competence of cat oocytes [42,43,44]. Hence, we suspect that addition of loose cumulus cells to IVM medium after vitrification-warming procedures may complement some post-vitrification deficiencies, but specific studies must be performed.

Immature oocytes can be vitrified directly after collection from ovarian follicles; thus, no special laboratory equipment is needed. Being “field friendly” is a crucial advantage of immature oocyte vitrification in the context of wildlife conservation. However, it is important to point out that immature oocytes are selected for cryopreservation based on their morphology and a ‘normal’ appearance does not guarantee maturational competence [18]. Our data and some published results show that degeneration rate is definitely higher in immature oocytes, as they still have to undergo maturation after warming, before being fertilized [2,3,39]. Performing in vitro maturation before vitrification, implies a big advantage of vitrifying only metaphase-II stage oocytes. These oocytes have already demonstrated the competence to complete nuclear maturation, which is pivotal to further development [6]. To perform in vitro maturation, however, laboratory equipment is necessary, a precondition hardly achievable in a field setting.

In the course of this study, morphologically normal embryos up to morula stage have been produced from oocytes vitrified in immature and mature nuclear stage with the use of ICSI. It should be emphasized that oocytes vitrified as immature showed significantly lower suitability for ICSI (10.4%) when compared to MII (61.5%) and control group (49.4%; *p* < 0.05). On the other hand, meiotic stage of vitrified oocytes did not affect cleavage rate (immature and in vitro matured—25% and 21.9%, respectively), although it was significantly lower than in the control group (57.4%; *p* < 0.05). 

In order to assess the developmental competence of examined oocytes, ICSI was implemented. Due to the premature exocytosis of cortical granules during cryopreservation, zona hardening occurs, leading to a significant reduction in fertilization rate when IVF method is used [17,45]. Moreover, due to cryoinjuries of the zona pellucida, polyspermic fertilization is a relatively common phenomenon [46] at least in humans, which was the reason to use IVF as a method of fertilization in experiment 1. Intracytoplasmic sperm injection is the accepted method used to bypass those negative consequences of cryopreservation, but at the same time, it should be noted that very costly equipment and highly trained personnel are needed to perform it.

The possibility of obtaining high fertilization rates (50%) utilizing ICSI to fertilize cryopreserved human oocytes was shown by Gook et al. [47]. Results reported by Kazem et al. [46] and Mavrides et al. [48] showed improvement in cleavage rate of oocytes subjected to ICSI compared to standard IVF in human and bovine oocytes, respectively. Pope et al. [25] were the first to confirm ICSI as a successful method for cat oocyte fertilization with the birth of a live kitten in 1998. Since then, many further studies have been reported [1,21,29,49,50,51,52] substantiating the applicability of this method for fertilization of fresh oocytes.

To date, only two published studies concern the ICSI of cryopreserved cat oocytes [5,6]. Pope et al. [6] did not show any statistical differences in cleavage rate between oocytes fertilized with the use of IVF and ICSI. However, due to a single replicate and low number of vitrified oocytes (17 in the experimental group and five in the control group) these results are not relevant in terms of the fertilization technique used, notwithstanding the extraordinary success of the birth of live kittens [6].

## 5. Conclusions

Although the vitrification procedure itself significantly reduced the meiotic competence of oocytes, their meiotic status before vitrification (immature vs. in vitro matured) did not influence fertilization and morula rates. The only parameter affected by vitrification was the rate of oocytes suitable for ICSI, which was significantly lower for immature oocytes.

Regardless of the meiotic status of vitrified oocytes, morphologically normal morulae were obtained. Vitrification of IVM oocytes seems to be a more effective way of preserving cats’ genetic material than is the vitrification of immature oocytes. It should be also mentioned that vitrification protocol for immature oocytes may be more applicable to the field conditions, since it does not require any particular equipment. However, it cannot be easily answered which meiotic status is more suitable for vitrification because it depends on several conditions. In case of a fully equipped lab, vitrification of matured oocytes may be the method of choice. Another limiting factor is the number of good quality COCs per feline ovary, which is less than five. A potential way to overcome these restrictions may be to bank primordial follicles or the ovarian cortex, unless the success rate of oocyte cryopreservation has not increased. Considering the current state of knowledge, cryopreservation of oocytes has still limited application to bank gametes of endangered feline species and thus requires more research.

## Figures and Tables

**Table 1:** Analyzed parameters related to oocyte maturation, fertilization, and embryo development with regard to exposure to cryoprotectants in vitrification solution.

Groups of COCs	Number of COCs	Maturation Rate % (*n*)	Cleavage Rate% (*n*)	Morula Rate % (*n*)
Toxicity test	47	56.8 (21)	38.1 (8)	14.3 (3)
Control	46	52.5 (21)	38.1 (8)	19.1 (4)

No significant differences were found between groups.

**Table 2:** Developmental competence of immature and mature cat oocytes vitrified by the cryotop method.

Groups of COCs	Number of COCs	Used for ICSI % (*n*)	Cleavage Rate % (*n*)	Morula Rate % (*n*)	Blastocyst Rate % (*n*)
Vitrified immature	154	10.4 (16) ^a^	25.0 (4) ^a^	25.0 (1) ^a^	0 ^a^
Vitrified mature	156	61.5 (96) ^b^	21.9 (21) ^a^	33.3 (7) ^a^	0 ^a^
Non-vitrified	233	49.4 (115) ^c^	57.4 (66) ^b^	66.7 (44) ^b^	24.2 (16) ^b^

Different superscripts within the columns represent significant differences (*p* < 0.05).
